# Accessing HIV services in Botswana: perspectives of men who have sex with men and other stakeholders

**DOI:** 10.1080/16549716.2023.2262197

**Published:** 2023-10-13

**Authors:** Kgosiekae Maxwell Matlapeng, Gbotemi Bukola Babatunde, Netsai Bianca Gwelo, Olagoke Akintola

**Affiliations:** a School of Public Health, University of Western Cape, Cape Town, South Africa; b Public Health Science Department, Institute of Health Sciences, Serowe, Botswana; c Graduate School of Professional Psychology, University of Denver, Denver, CO, USA

**Keywords:** Botswana, discrimination, healthcare services, HIV/AIDS, HIV/AIDS policies, HIV services, key population, men who have sex with men, MSM

## Abstract

**Background:**

Men who have sex with men (MSM) represent an increasing number of new HIV infections in Botswana. Many in Botswana still hold discriminatory views against people who engage in same-sex practices. While it is well documented that stigma and discrimination undermine efforts to prevent HIV, the literature about the perception and experiences of discrimination against MSM in accessing HIV services in Botswana is scant.

**Objective(s):**

In this study, we sought to explore the perception and experiences of discrimination against MSM to improve access to HIV services and reduce discrimination against this marginalised population.

**Methods:**

We employed a descriptive qualitative design using purposive sampling to recruit 20 MSM and 12 stakeholders (six policy-makers and six service providers) involved in implementing HIV/AIDS interventions in Botswana. We conducted semi-structured interviews with participants. All data were audio-recorded, transcribed, translated into English and analysed using thematic analysis.

**Results:**

The findings of this study show that MSM experience discrimination at the policy, healthcare system and community levels, which undermines their ability to seek HIV services. The participants reported that MSM are excluded from HIV policies and targeted HIV services. They reported a scarcity of trained personnel, poor access to HIV information, condoms, lack of treatment services targeted at MSM and negative attitudes directed towards MSM by service providers and other users. The participants also reported that they are excluded from community interventions and experience negative attitudes from community and family members.

**Conclusion:**

Discrimination against MSM undermines the ability of HIV interventions to address MSM sexual health needs. The findings indicate the importance of enabling MSM to overcome discrimination to seek HIV services. Also, there is a need to further explore discrimination against MSM by service providers within the healthcare settings and ways to improve their understanding of male same-sex practices.

## Background

Stigma and discrimination towards men who have sex with men (MSM) undermine interventions designed to reduce HIV infections and engage people in HIV treatment, care, and support programmes [1]. MSM are among the most marginalised and at-risk populations and may experience multiple layers of stigma due to their sexual orientation, gender identity and/or race/ethnicity in addition to their HIV status [1]. Goffman defines stigma as an attribute that is discrediting, while discrimination is the unjust, prejudiced distinction between people based on groups, classes or categories to which they belong [2]. People can be discriminated against based on age, race, gender, religion, sexual orientation and disability among others [2]. Goffman argues that stigma (the negative stereotype) often leads to discrimination (the behaviour that results from the negative stereotype) [2]. Grossman and Stangl stated that discrimination is fuelled by myths of casual transmission of HIV and pre-existing biases against certain groups, sexual behaviours, fear of illness and death [3].

In sub-Saharan Africa (SSA), MSM are less visible due to stigma and discrimination [4]. Discrimination against MSM is associated with the fear of seeking healthcare, fear of disclosing one’s sexual orientation and verbal abuse [5]. Li et al. identify stigma and discrimination as primary obstacles to effective HIV prevention among MSM [6]. In addition, social and health inequities surrounding HIV/AIDS resulting from stigma and discrimination are significant barriers to effective HIV control [7]. According to Chambers et al., negative social response towards HIV/AIDS also exacerbates the pandemic [7].

In Africa, homosexuality is viewed as immoral, unacceptable and alien to traditional values that impede lineage continuity [8]. Many African countries still criminalise same-sex practices. Of the 72 countries that criminalise same-sex practices worldwide, 32 are in Africa, where punishments range from imprisonment to death penalty [8–10]. In Namibia and Malawi, same-sex practices are punishable by civil imprisonment [9]. While homosexuality is outlawed in Nigeria and laws have been tightened over the years, it is only punishable by death in the northern parts where there is Sharia Law [10]. As a result of the intolerant attitudes and concomitant penalties, people who engage in same-sex practices have become the prime target of religious and political persecution, thereby increasing homophobic rhetoric [11].

Male same-sex practice is a taboo subject in Botswana and is seen as alien to Africa [12]. The Botswana Penal Code states that ‘any individuals who commit acts of gross indecency with another person are guilty of an offence’ (p 67) [13]. A study conducted in 2002 by the Global Fund found that Botswana’s HIV laws and policies impede access to HIV services and HIV intervention for key populations such as sex workers, homosexuals and people in rural areas [14]. After this report, the government introduced policy amendments that led to the implementation of the National Strategic Framework II and the Botswana National Operational Plan for Scaling up HIV Prevention [15]. This policy reform led to the implementation of targeted HIV programming and interventions such as condom promotion and STI screening for sex workers and truck drivers. However, the policy reforms excluded MSM. A study conducted in 2016 by Matlapeng et al. indicates that Botswana’s HIV policies do not have specific provisions that address the sexual health needs of MSM population [16]. However, same-sex practice was decriminalised in 2019, following a High Court ruling that Sections 164(a)–164(c) and 167 of the Botswana Penal Code that criminalises same-sex practices are discriminatory and unconstitutional [17].

Despite the positive developments in the policy and legal arenas, MSM are not yet recognised as a key population within the national HIV response [18]. This limits the implementation of targeted HIV interventions for male same-sex practice. In addition, MSM continue to experience challenges within the healthcare system and within their families and local communities. These include verbal and physical abuse, sexual violence and threats of blackmail and exposure [18]. The lack of governmental support and commitment to implementing targeted HIV services for MSM remains a significant barrier to accessing specialised services for this group [18].

Botswana is one of the most affected countries by HIV in the Southern African region, with a population estimated at 2.92 million people, 18% of whom live with HIV [19]. The 2012 Behavioural Surveillance Report of the 12 health districts in Botswana estimated the population of MSM at 2006, with an HIV prevalence of 14.8% [19]. The report also found an HIV prevalence estimate of 13.1% among the 781 MSM in two towns, namely Francistown and Gaborone [19]. This is similar to the HIV prevalence of 12.9% in the general population among males aged 20–30 years in 2008 [19]. The sample was young (MSM) with a mean age of about 23 years and HIV incidence of 3.6% which is higher than the national average of 1.35% and higher than among pregnant women estimated at 2.7% [19]. The STI prevalence among this group was estimated at 10%, which is more than the general population [19]. This calls for concern that HIV prevalence among this group may be higher, hence more studies are needed to collect data on HIV prevalence among MSM in Botswana.

According to BMoH, alcohol abuse and limited information on HIV infection relating to male same-sex practice are some of the major factors that increase infection among this group [19]. The BMoH estimates that 65.1% of the participants were unaware that anal sex is associated with an elevated risk of HIV acquisition, and 46.5% reported not having received any HIV-related information on male same-sex practice [19]. The Global Fund points out that as of 2016, Botswana lacked specific strategies that address the health needs of MSM and that HIV interventions are provided in the context of the heterosexual population [20]. Due to the legal framework in Botswana that discriminates against male same-sex practices, MSM were characterised as a non-priority population and not included in the country’s definition of targeted key populations [20].

A study on human rights violations against MSM in Southern Africa found that 58.6% of participants in Botswana experienced human rights infringements, including blackmail, stigmatisation and discrimination [4]. These acts undermine the effectiveness of HIV and sexual health programmes and the goal of reaching the MSM population [21]. Despite the myriad of challenges confronting MSM, research about the perception and experiences of discrimination against MSM accessing HIV services in Botswana is scant. Therefore, this study explored the perspectives of policy-makers, service providers and MSM about the discrimination against MSM in the country. The findings from this study could contribute new knowledge and inform the planning of evidence-based programmes to reduce discrimination among MSM. In addition, the findings could inform the enactment of new HIV/AIDS policies to increase access to HIV services and reduce discrimination among MSM.

## Objective

Discrimination undermines the effectiveness of HIV interventions, especially among key populations such as MSM. Yet, no study has sought to understand the perception and experiences of discrimination against MSM in accessing HIV services. The purpose of this study was to explore perception and experiences of discrimination against MSM in Botswana.

## Methods

### Study setting and context

This study was conducted among MSM living in Gabarone, the capital city of Botswana, who accessed services from the Centre for Men’s Health (CMH). The CMH is a non-governmental organisation that provides one-stop shop services to MSM. The services include HIV services, psychosocial support, and legal advice. The CMH is also an affiliate of the Human Rights Office (Ditshwanelo), which advocates for the promotion and protection of human rights in Botswana irrespective of gender, ethnicity, religion, sexual orientation, social status and political affiliation. The CMH has a trained and accredited psychologist who provides psychosocial support and counselling services to MSM, especially on serious issues such as acceptance, disclosing their sexual identity, suicide, coping with stigma, discrimination and family issues.

### Study design

This study adopted a qualitative research design. Qualitative research design is deemed appropriate because it explores the lived experiences of MSM and enables the researcher to capture rich information [22,23].

### Sampling procedure and study participants

We used a purposive sampling technique to select 32 participants [24]. The study involved three different groups of participants: policy-makers, service providers and MSM. Policy-makers and service providers were selected because they play significant roles in planning and implementing HIV testing and treatment programmes. Their experiences in dealing with MSM were deemed critical for exploring stigma and discrimination among MSM related to their access to HIV services. Thus, representatives from the public health facilities (Botswana Ministry of Health, BMoH) and non-governmental organisations were selected. We interviewed two policy-makers, four facility managers and six service providers.

MSM were selected to provide information on their perception and experiences of accessing HIV services within the Botswana healthcare system. To gather rich data, a homogeneous sample (MSM) was drawn, as suggested by Krueger [25]. Homogeneous sample was used because the sample (MSM) has a similar or common trait; hence, the sample was used to understand and describe a phenomenon under study. Also, the research questions addressed the specific characteristics of the participants under study. The CMH assisted with the recruitment process by providing a list of 39 potential participants. Only participants who had accessed or attempted to access HIV services through the government healthcare system were recruited. The CMH also provided support by engaging a counsellor through the Human Rights Office to provide psychosocial support when needed. The study selected 20 participants who met the inclusion criteria and were available and willing to participate. The principal investigator [KMM] met with the participants and provided detailed information about the purpose of the study and the counsellor’s roles. No incentive or remuneration was provided to participants as part of the recruitment process. The following inclusion criteria were used in selecting MSM: Participants must

1. be 18 years and above
2. engage in same-sex practices
3. have access to services from the CMH
4. have accessed or attempted to access services from government healthcare facilities.

### Data collection procedure

Data for the study were collected using semi-structured interviews, which allowed for the collection of in-depth information [26,27]. Semi-structured interviews were deemed appropriate as they allowed more flexibility in the interview schedule as the participants have more of an influence over the direction of the conversation. Participants can choose a time that works for them to reschedule the interview and this can increase the willingness of participants to agree to the interview. Also, it allows the participants to open up about sensitive issues and offers less chance for moderator bias than in focus group settings [27,28]. The interviews were guided by an interview schedule, which included mainly open-ended questions. The themes covered included: demographic information of MSM, experiences of accessing healthcare services, experiences of stigma and discrimination. The interview schedule was developed based on themes obtained from the review of literature on stigma and discrimination among MSM, health-seeking behaviours and access to healthcare services (HIV testing and treatment).

Individuals who agreed to participate were provided with information sheets and informed consent forms. They were encouraged to read the information sheet and ask questions. Thereafter, we sought and obtained written informed consent from each participant, assuring confidentiality and anonymity of the information collected. Also, permission to use a digital recorder and take field notes was obtained from all the participants. One of the authors (KMM) conducted interviews in English and Setswana, depending on the participants’ preferences. However, most of the participants responded both in English and Setswana once the interviews commenced. The interviews were conducted from April to May 2016, appointments were scheduled to suit the study participants, and follow-up appointments were scheduled to collect further information and seek clarity where necessary. Interview sessions lasted between 35 and 60 min each and were conducted at the CMH for MSM, while policy-makers’ and service providers’ interviews were conducted at their respective offices. Data saturation was achieved after 12 interviews, and eight additional interviews were conducted to ensure no new relevant information emerged. For policy-makers and service providers, saturation was reached after interviewing six participants.

### Data analysis

The data were transcribed verbatim, while data in Setswana were translated into English by the PI. We conducted data analysis following the six steps of thematic analysis suggested by Braun and Clarke [29]. The transcripts were read and reread to gain familiarity with the content and were checked against the recordings to confirm or correct any errors. The researchers identified and systematically coded relevant features of the data. A matrix was developed to categorise and compare data. This helped to identify common and divergent responses to the key questions. The data was categorised manually into themes generated based on the theory of access to healthcare services by Aday and Andersen [30]. Participant responses were categorised under appropriate themes. Within each of these sub-categories, responses were compared across participants, and themes were refined.

### Ethics

The study received ethical clearance from the Human Social Sciences Research Ethics Committee of the University of KwaZulu-Natal, South Africa (HSS/1726/015D). The Health Research and Development Division of the Botswana Ministry of Health reviewed the IRB and provided ethical approval and permission to conduct the study (HPDME 13/18/1 X (100)). In addition, gatekeepers’ permission was obtained from health facilities and NGOs. The study sought and obtained written informed consent from each participant after the interviewers provided a detailed explanation about the study, assuring confidentiality and anonymity of the information collected. To ensure confidentiality and anonymity, the study used pseudo-names to ensure that participants’ names are not linked to the transcripts. The interviewers obtained permission from the participants to conduct the audio recording of the interviews. Also, data are stored in password-protected folders, and hard copies are kept in locked cabins accessible to the principal investigator only. The data set will be destroyed after 5 years.

## Results

### Demographic profile of the participants (MSM)

Of the 20 participants, the median age was 26.5, standard deviation 5.7 and mean 27.1 years. The majority of MSM had tertiary (10) and secondary (7) education, and 3 had junior secondary education, 13 were employed, unemployed 6 and one participant was a student. There were 16 participants who identified as ‘she’ male (receptive partners), while 4 identified as insertive partners. Also, 16 participants identified as gay and did not have children, while 4 identified as bisexuals and having children. The majority of participants indicated that they were living with friends or other relatives, while two were cohabitating with their female partners. Data on HIV status was not collected; however, eight participants disclosed their status (HIV+) and 12 had unknown status. At the time of data collection, MSM participants were accessing HIV services at the CMH and government facilities.

### Discrimination against MSM

Three main themes and associated sub-themes that contributed to discrimination against MSM were identified. They include HIV/AIDS policy-level influences, health system-level influences and community-level influences (see Table 1).

**Table 1.** Themes relating to stigma and discrimination among MSM.

Main themes	Sub-themes
HIV/AIDS policy-level influences	● Limited recognition of MSM in HIV/AIDS policies ● Exclusion of MSM from targeted HIV interventions
Health system-level influences	● Shortage of trained personnel ● Limited access to HIV information, condoms and treatment ● Verbal abuse by service providers and other healthcare users.
Community-level influences	● Exclusion from community health interventions ● Negative attitudes by community members and family rejection

#### HIV/AIDS policy-level influences

Policy-level factors that participants identified as contributing to discrimination include limited recognition of MSM in HIV/AIDS policies and exclusion of MSM from targeted HIV interventions.

#### Limited recognition of MSM in HIV/AIDS policies

MSM participants acknowledged the existence of Botswana HIV/AIDS policies such as the National Health Policy, the Revised National Policy on HIV/AIDS and the National Strategic Framework for HIV/AIDS. These policies addressed the health needs of the general population and prioritised some at-risk populations such as sex workers, children, truck drivers and women. However, MSM participants noted that the national HIV policies excluded MSM from the populations identified as being at risk of HIV infection. MSM stated that there were no policies that advocated for their sexual health needs and that this amounted to discrimination. Their exclusion from policies was cited as a major barrier to accessing healthcare services. MSM participants further indicated that national HIV policies were silent on issues such as HIV interventions, coordination of resources and key priorities for MSM to reduce transmission. One participant noted:

The HIV policy does not specifically address the service needs for same-sex practices and excludes us from targeted HIV interventions. (24 year old MSM)

The HIV testing representative also indicated that the exclusion of MSM in HIV policies is a barrier to service provision for this population. She highlighted the link between the prevailing legal framework and access to HIV services among MSM.

It affects access to services in a big way because the legal framework is not in their favour, then their access to testing and treatment services is limited. That is why we are trying to capacitate service providers (NGOs) so that while the legal system is not conducive, they should be able to facilitate access to services. (HTC policy-maker)

There is an acknowledgement of the limitations the legal framework imposes on the health policy since the country’s policies are developed in line with the current legal framework that does not recognise MSM. However, the HTC policy-maker noted the health departments’ willingness to address this problem. According to the participant, service providers (NGOs) are capacitated to provide the required healthcare services for this population. The HTC policy-maker pointed out that the department collaborates with local and international donor partners to provide capacity building of NGOs (service providers) to address the sexual needs of this population. Also, through the Ministry of Health, the donor partners provide funds to NGOs to provide targeted services to MSM.

#### Exclusion of MSM from targeted HIV interventions

MSM participants noted that the BMoH provides specific targeted HIV interventions to meet the health needs of different at-risk populations. They, however, noted that there are no interventions that target MSM despite the fact that they are also an at-risk population. Instead, MSM access HIV services from the mainstream programmes for the general population. MSM who participated in the study felt judged, intimidated, uncomfortable and unprotected from HIV because their sexual health needs are not considered in HIV programmes that target the general population. MSM participants indicated that:

Many of us (MSM) do not bother to go to the clinic anymore because sometimes they scream at us. We know we will be unfairly treated because they will ask us why do you sleep with another man. (24 year old MSM)

Most of our clients [MSM] mentioned that they are sent away when they request to test a couple. Most people [service providers] are guided by societal norms and that is why society has not accepted us [MSM], they see us as cursed or less human beings. (37 year old MSM)

Both participants, the BMoH and NGOs, indicated that HIV services are provided to MSM without considering their unique health needs. They acknowledged that the absence of targeted interventions for MSM increased the risk of transmission among this group. The service providers (BMoH and NGOs) reiterated that the provision of HIV services based on the heterosexual population context infringes on sexual minorities' basic human rights to access healthcare services.

The Ministry of Health provides services on the basis of an all-inclusive policy without targeting particular populations. (HTC policy-maker)

The recent HIV policy review has recognised the need to deliver HIV services without discrimination while acknowledging the existence of drivers of HIV infection (target populations such as MSM) that require special attention. However, nothing has been done to provide resources and infrastructure to improve service provision to MSM.

#### Health system-level influence

Factors that participants identified as contributing to discrimination against MSM within the healthcare delivery system include the shortage of trained personnel, limited access to HIV information, condoms and treatment, and verbal abuse by service providers and other healthcare users.

#### Shortage of trained personnel

MSM participants in our study pointed out that the BMoH collaborated with NGOs to provide services to some of the groups identified as the most at-risk populations, such as truck drivers and sex workers. However, there are no dedicated health personnel to attend to their unique sexual health needs. MSM explained that service providers from BMoH and NGO facilities lack relevant information on issues relating to male same-sex practices and that this undermines the quality of services they provide to them. One participant (MSM) stated:

The counsellor kept repeating the same information and kept quiet for a long time just to kill time. (37 years MSM)

Many of the HIV testing service providers (BMoH and NGOs) corroborated the assertions made by the MSM who participated in the study. For example, the service providers stated:

It is difficult to offer them (MSM) services, so we sometimes randomly refer them to organisations dealing with HIV interventions even though we are unsure they will receive the proper services. (HIV testing service provider)

BMoH and NGO service providers acknowledged their responsibility to provide services to MSM. However, they lack the knowledge and skills (competencies) to deal with issues specific to MSM.

#### Limited access to HIV information, condoms and treatment

MSM participants mentioned that the BMoH provides HIV information, condoms, water and oil-based lubricants, including female condoms nationwide for the heterosexual population. Although MSM acknowledge the availability of condoms for heterosexual population and female condoms in healthcare facilities, they preferred male same-sex condoms to be available. The participants (MSM) stated that information relating to HIV is regulated by the BMoH and that information is often directed at the heterosexual population. MSM participants mentioned that there is no information on male same-sex in healthcare facilities. On the other hand, these interventions and innovations are not provided in healthcare facilities for MSM. Many of them indicated that male same-sex condoms and their lubricants are only sold in pharmacies and are unaffordable. MSM participant stated that:

They (service providers) talk about preventing STIs by using condoms, but they do not provide us (MSM) with male same-sex condoms and lube. They provide for heterosexual people only. (26 year old MSM)

Similarly, MSM participants stated that pre-exposure prophylaxis (PrEP) and post-exposure prophylaxis (PEP) are available to populations identified as most at risk of HIV infection, such as healthcare service providers and rape survivors. However, MSM participants noted that they should be provided with PrEP and PEP to prevent HIV infection due to their unique sexual health needs.

We also need more information on HIV prevention because more studies show good results on prevention treatment. So, in my opinion, and looking at the risk of anal sex, it is prudent to provide treatment (PrEP and PEP) to gay people. (37 year old MSM)

#### Verbal abuse by service providers and healthcare users

MSM participants explained that they experience abuse from BMoH service providers and healthcare users. For example, they noted that BMoH service providers commonly use derogatory and demeaning words like ‘matanyola’, which refers to male same-sex practices among prison inmates, to refer to them. Similarly, some of the MSM participants explained that some clients use words meant to demean or insult them, such as calling them ‘inbreeds’ or claiming that they are possessed by evil spirits. One MSM participant indicated that:

The nurse said to me: ‘how did I get an anal infection’. I told her I had unprotected sex with a man, and she became so angry and scolded me for sleeping with a man. I received a long lecture that it was immoral to have sex with a man and that I should be delivered from my demons. I felt insulted and not respected. (23 years old MSM)

An important observation made by MSM participants was that the services they received in government facilities were different from those received from private healthcare centres and NGOs. MSM participants observed that they are likely to be verbally abused by service providers at government facilities than at private health facilities and NGOs. Similarly, MSM participants observed that nurses in public healthcare facilities were the typical culprits. They often use abusive language more frequently than the doctors and also frequently violate their privacy. An MSM participant indicated that:

I told her (nurse) that I had piles, and she responded, ‘*oooh ba go jele matanyola’*, [a derogatory word meaning ‘he had sex with a man’]. That was offensive to me. (22 years old MSM)

BMoH service providers also reiterated that in some instances, MSM are treated unfairly or verbally abused by colleagues. Some service providers (BMoH) mentioned that some of their colleagues view men who identify as gay or who engage in same-sex practices as having a mental disorder. They noted that people perceive MSM as outcasts because of their behaviour that deviates from social norms. Service providers (BMoH) also acknowledged that they display prejudice towards MSM due to a lack of understanding of their sexual health needs and cultural, religious and social norms. Some of the service providers indicated that:

At times, they [MSM] get on your nerves with their woman-like attitudes, and it is irritating to deal with a man who thinks he is a woman. It takes a lot to help them. So if you are having a bad day at work, you explode with anger. (HIV testing service provider)

Service providers with conservative values view them [MSM] as cursed. They will start quoting the Bible that it is wrong to have male same-sex, and most of the time it ends badly as they [MSM] will respond with anger to them. (HIV testing service provider)

Service providers (BMoH), particularly those with conservative religious or cultural values, constitute major barriers to accessing services for MSM as they are either verbally abused or embarrassed when they attempt to access services.

#### Community-level influence

Factors that were identified as contributing to discrimination against MSM at the community level include exclusion from community health interventions and negative attitudes by community members and family rejection.

#### Exclusion from community interventions

MSM participants indicated that government initiatives to normalise HIV in communities are rolled out through community-led interventions. These interventions include community mobilisation, HIV testing, condom promotion and HIV information through roadshows and edutainment. They are coordinated by the District Multi-Sectorial AIDS Committees (DMSAC). MSM participants stated that community structures such as civil societies, NGOs and Village Development Committees engage communities through the *Kgotla* system to address HIV issues. A *Kgotla* is a traditional court in Botswana and the custodian of cultural beliefs where issues of national interest such as community initiatives and development plans are discussed. However, MSM indicated that the *Kgotla* system upholds traditional laws that do not allow homosexuality. Therefore, MSM organisations and advocacy groups are excluded from consultations and implementation of community health interventions. According to one MSM participant,

One cannot advocate for male same-sex practice sexual health needs in the *Kgotla* because it is against customary laws. It does not accept homosexuality. (29 year-old MSM)

Both the BMoH and NGO service providers reiterated that MSM are excluded from community planning and involvement in HIV interventions. They mentioned that community consultations and funding of interventions focus on heterosexual relationships.

The Ministry of Health and the district’s health teams have put all the means to reach communities through programmes. However, such planning or implementation of interventions does not take into account diverse groups like MSM. (NGO HIV testing service provider)

#### Negative attitudes by community members and family rejection

The majority of MSM participants illustrated that they experience negative attitudes from the community, including family members, because of their sexual orientation. They indicated that they constantly encounter community hostility, such as insults and physical confrontations. MSM participants noted that they are reluctant to access community interventions for fear of public humiliation. Some of the MSM participants from families with conservative values stated that they were disgraced and scolded for bringing their family name into disrepute because of their sexual orientation. MSM participants stated that:

During a family meeting, my father yelled at me that I was not born gay and that none of us in our family lineage and my mother’s family is gay. He said I was tormented by evil spirits that needed to be exorcised. (23-year-old MSM)

My family disowned me after they learnt that I was sleeping with other men. I was sick for a very long time before I started treatment. When they learnt that I was involved with other men, they disowned me, I moved from the village to the city. (37 year old MSM)

MSM participants who disclosed their sexual orientations to their families experienced negative attitudes and stigmatising reactions which highlights the extent to which cultural and religious biases directly influence people’s views and opinions of MSM.

## Discussion

This study provides insight into factors that contribute to discrimination against MSM while accessing HIV services in Botswana. Both MSM participants and service providers noted that there is limited recognition of MSM in HIV policies and exclusion from targeted HIV interventions. The findings reveal the absence of provisions to address the sexual health needs of MSM in national HIV policies. As a result, the MSM population are excluded from targeted services designed for key populations, leading to discrimination while accessing HIV-related services. Our findings on the limited recognition of MSM in HIV policies and exclusion from targeted services support previous studies in sub-Saharan Africa (SSA). These studies show that the criminalisation of same-sex practices undermines HIV interventions among this population [31–33].

According to Abara and Abara, many countries in SSA have failed to address the sexual health needs of MSM in national HIV/AIDS programmes [32]. This is substantiated by the UNAIDS report, which found that about 70% of countries globally criminalise and exclude MSM from national HIV planning, especially in low- and middle-income countries (LMICs) [34]. Our findings suggest that limited attention given to MSM and exclusion from targeted services could increase the risk of HIV exposure among MSM. Hence, HIV infection among MSM could go unaddressed and impede access to HIV services. Previous findings show that barriers at policy level play a significant role in poor health seeking behaviour, access to healthcare services, social alienation and poor mental health outcomes among MSM [35–37]. Based on these observations, this study underscores the importance of revising legal and policy frameworks to address the sexual health needs of MSM.

Our findings highlight the lack of key provisions that address HIV infection among MSM within Botswana’s HIV policies. Failure to accommodate the sexual/health needs of people who engage in same-sex practices within HIV policies is a major barrier to improving health outcomes including HIV response initiatives. This is likely to impede access to HIV services, increase their vulnerability to infection and infringe on their fundamental human rights. In addition, the failure to include MSM in HIV policies and exclusion from targeted HIV interventions violates the NSF II 2010–2016, the UN Human Rights Charter (UNHRC) and the International Covenant on Civil and Political Rights (ICCPR). These documents spell out the importance of human rights in fighting HIV. These policies stipulate that HIV interventions should provide non-discriminatory welfare services to everyone and ensure equal access to healthcare and psychosocial support services to the entire populace [38]. Therefore, policy-makers need to review the Botswana national HIV/AIDS policies to address MSM sexual health needs. The Botswana HIV policies should emphasise equity and a human rights approach to providing comprehensive healthcare services for MSM.

Our study shows that the healthcare system in Botswana grapples with the shortage of skilled personnel, targeted HIV information, male same-sex condoms and lubricants. Participants stressed that limited targeted resources for MSM in public healthcare settings contribute to discrimination, which impedes access to healthcare services. Previous studies have shown that the lack of resources within healthcare settings inhibits health service utilisation among MSM in low- and middle-income countries [39,40]. There is a need to prioritise comprehensive HIV interventions for MSM. Similarly, prior findings have shown that a lack of skilled personnel or limited skills and knowledge in addressing MSM alienate them from accessing healthcare services and negatively impacts the provision of appropriate risk reduction interventions [36,41]. Therefore, this study calls for resource mobilisation to provide targeted services to MSM. For example, studies in Kenya have demonstrated that providing service providers with targeted HIV knowledge and skill could improve the provision of services and reduce homophobic attitudes towards MSM [42].

MSM are likely to be unaware of the risk factors associated with male same-sex practices and unable to make informed decisions to prevent HIV infection due to the limited HIV sensitisation for this population. A study conducted in Tanzania by Magesa et al. found that inadequate HIV prevention programmes, particularly awareness interventions, lead to poor health-seeking among MSM [36]. Besides insufficient information, other factors such as socio-economic status, limited agency and power could potentially influence MSM health decision-making. Therefore, we recommend that subsequent studies focus on investigating the above factors as they are important predictors of healthy decision-making to prevent HIV infection. In addition, it is imperative to build strategic alliances between the government and organisations at the local, regional and international levels to increase targeted resources and address MSM sexual health needs. There should be a synergy of HIV interventions among NGOs dealing with key populations to maximise resource sharing at the local level. There might be a need to strengthen and fund NGOs working with MSM to expand and sustain service provision. Also, service providers should be trained to provide the required specialised HIV services in a safe, conducive and friendly environment for MSM. The government could collaborate with regional and international donors to improve resource mobilisation. These alliances should include partnerships to share or transfer technology and information and maximise resource mobilisation and utilisation.

This study shows that same-sex practices remain highly unacceptable in Botswana communities. Our study revealed that MSM experienced social stigma from the community and their families. Cultural and religious beliefs are fundamental drivers of these negative attitudes and behaviours towards MSM. The MSM who participated in the study noted that community rejection limits their access to healthcare services. Therefore, educating the communities on the need to destigmatise same-sex practices should be included in HIV awareness programmes.

## Strength and limitations

A crucial strength of the study was that we collected data using semi-structured interviews, which provided rich information on the experiences of discrimination against MSM. Semi-structured interviews allowed us to explore factors contributing to discrimination through the lens of MSM. The use of qualitative methodology poses some limitations to the study findings. This study used purposive sampling to recruit a small sample. Hence, the findings of this study cannot be generalised to other locations as the data represents the views of a limited number of MSM. Nevertheless, the results are valuable as a source of insight into the views of MSM. Second, biases and socially desirable responses are unlikely to be eliminated due to the sensitivity of the topic. Therefore, a mixed methods study is required to provide an in-depth description of factors associated with discrimination against MSM in Botswana.

## Conclusion

The findings of this study shows that discrimination continues to impede HIV services utilisation among MSM. Therefore, policy reform should classify MSM as a key population and include them in HIV interventions. Similarly, resource mobilisation is crucial as the government should engage with partners to provide targeted interventions and services that promote the fundamental human rights of sexual minorities. These interventions should include a political commitment to providing the required resources to develop sustainable HIV interventions that reduce discrimination against MSM. Additionally, programmes aimed at destigmatising same-sex practices and providing accurate information that challenges cultural and religious prejudice against MSM through community awareness is crucial to addressing the health needs of this population. These considerations are critical to reducing social hostility and new HIV infection.

## Paper context

Globally, stigma and discrimination undermine the effectiveness of HIV interventions, particularly among men who have sex with men (MSM). This study reveals that MSM in Botswana experience social discrimination at the policy, healthcare system and community levels. These undermine the ability of MSM to access HIV services. Therefore, it is important to prioritise policy and structural reforms that promote specialised HIV services for MSM to increase service uptake among this key population.
